# An Assessment of the Validity and Reliability of the Pediatric Child Health Utility 9D in Children with Inflammatory Bowel Disease

**DOI:** 10.3390/children8050343

**Published:** 2021-04-27

**Authors:** Naazish S. Bashir, Thomas D. Walters, Anne M. Griffiths, Wendy J. Ungar

**Affiliations:** 1 Program of Child Health Evaluative Sciences, The Hospital for Sick Children Research Institute, Toronto, ON M5G 0A4, Canada; naaz.bashir@sickkids.ca; 2 Division of Gastroenterology, Hepatology and Nutrition, The Hospital for Sick Children, Toronto, ON M5G 1X8, Canada; thomas.walters@sickkids.ca (T.D.W.); anne.griffiths@sickkids.ca (A.M.G.); 3 Institute for Health Policy, Management and Evaluation, University of Toronto, Toronto, ON M5T 3M6, Canada

**Keywords:** health utilities, CHU9D, HUI, Crohn’s disease, ulcerative colitis, pediatrics, economic evaluation, health state preferences

## Abstract

Health utilities relevant to children are lacking, compromising health funding and policy decisions for children. The Child Health Utility 9D (CHU9D) is a recently developed preference-based health utility instrument designed for use in children. The objective was to examine the validity of the CHU9D in a cohort of 285 Canadian children aged 6.5 to 18 years of age with Crohn’s disease (CD) and ulcerative colitis (UC), (collectively inflammatory bowel disease (IBD)). The correlation and agreement between paired CHU9D and Health Utility Index (HUI) assessments were determined with Spearman coefficients and Bland–Altman levels of agreement. Total and domain utilities were calculated for the CHU9D using Australian adult and youth tariffs. Algorithms for HUI2 and HUI3 were used. Domain correlations were determined between domains with expected overlap between instruments. In CD and in UC, correlations between CHU9D, HUI2, and HUI3 utilities ranged between 0.62 to 0.67 and 0.67 to 0.69, respectively (*p* < 0.05). CHU9D utilities were lower using youth tariffs compared to adult tariffs. A large range in health utilities suggested a heterogeneous quality of life. The CHU9D is a good option for preference-based utility measurement in pediatric IBD. Additional research is required to derive pediatric tariffs to conduct economic evaluation in children.

## 1. Introduction

Economic evaluations, such as cost–utility analyses, are increasingly used as part of the health technology assessment of new interventions for public or private payer reimbursement across the globe. Quality-adjusted life years (QALYs), an output of economic evaluation, are a measure weighting the length of life by health-related quality-of-life, expressed as health utilities. Health utilities represent an individual’s preference for a health state and are typically scored between the anchors of 0, representing death, and 1, representing perfect health (negative values are also possible to indicate states worse than death), and can be determined using generic health utility instruments. Generic health instruments are questionnaires that comprise both a classification system and a valuation algorithm based on multi-attribute utility theory [1]. The classification system describes domains of health-related quality of life according to severity levels. Preference-based generic health utility instruments allow for a comparison of health-related quality of life across diverse patient populations. These instruments have been developed and validated in adult populations but have been understudied in children [2,3].

In the United Kingdom (UK), the National Institute for Health and Care Excellence (NICE) has recommended the use of the EuroQol 5-Dimensions questionnaire (EQ-5D), but has stated that “consideration should be given to alternative standardised and validated preference-based measures of health-related quality of life that have been designed specifically for use in children” [4]. The Canadian Agency of Drugs and Technologies in Health (CADTH) recommends the use of health preferences based on a generic classification system such as the EQ-5D, Health Utilities Index (HUI), and the Short Form 6-Dimensions (SF-6D) but fails to specify special considerations for a pediatric population [5]. Guidelines have not recommended the use of a particular health utility instrument in children as sufficient data have not been generated to evaluate existing adult tools. Having standardized generic health utility instruments for children will facilitate the economic evaluation of interventions for children.

### 1.1. The Child Health Utility 9 Dimensions

The Child Health Utility 9 Dimensions (CHU9D) is a pediatric-specific preference-based measure developed in 2009 [6,7]. This instrument features a descriptive classification system exclusively developed with children to reflect health states relevant to child health [8,9]. The CHU9D covers nine dimensions: Worried, Sad, Pain, Tired, Annoyed, Schoolwork, Sleep, Daily routine, and Activities. Each dimension is represented by five levels such as Not, A little bit, A bit, Quite, and Very. Utility weights, or tariffs, for a selection of health states described by the system, were obtained from samples of the Australian adult population or from Australian adolescents using a best–worst scaling method [10,11]. The instrument focuses on the current health state (present-day recall). The CHU9D takes approximately 5 min to complete and is suitable for self-completion, by the child or with assistance, in children aged 7 to 17 years. A parent proxy version is also available. The CHU9D has been previously studied in mental health, in dental health, and in cerebral palsy [12,13,14]. It has also been used in mapping studies with other non-preference-based quality-of-life instruments in an attempt to generate health utilities [15,16,17,18,19,20].

### 1.2. The Health Utilities Index

The HUI refers to both the HUI Mark 2 (HUI2) and HUI Mark 3 (HUI3) which are multi-attribute, preference-based, generic health classification systems used to generate health utilities [21]. Both the HUI2 and HUI3 are currently commonly used to generate pediatric utilities using a different set of domains. Therefore, comparing the CHU9D to both the HUI2 and HUI3 is warranted The HUI has been established for use in adult and pediatric populations [22]. The HUI2 classification system includes seven attributes—Sensation, Mobility, Emotion, Cognition, Self-Care, Pain and Fertility—each with 3 to 5 levels [21]. The HUI3 classification system includes eight attributes—Vision, Hearing, Speech, Ambulation, Dexterity, Emotion, Cognition and Pain—each with 5 or 6 levels of ability/disability [21]. The HUI questionnaire can be self-completed or interviewer-administered in adults and in children over 8 years of age and takes approximately 3–10 min to complete [22]. Proxy versions are available for parents to report on preferences for health states of children as young as 5 years of age. The HUI was developed by having participants rate states on a visual analog scale and a standard gamble chance board [21]. The HUI is available with a usual health, one-week, two-week, or 4-week health recall period. The same HUI questionnaire can be used to calculate the single and multi-attribute utilities for the HUI2 and the HUI3 by using the respective valuation algorithms. The HUI has been used in numerous patient populations and healthy populations across several age groups [22].

### 1.3. Inflammatory Bowel Disease

Inflammatory bowel disease (IBD) represents a group of chronic gastrointestinal disorders such as Crohn’s disease (CD) and ulcerative colitis, in which areas of the gastrointestinal tract become inflamed and ulcerated [23]. Common symptoms of IBD include abdominal pain, diarrhea, and weight loss [23]. Growth may also be affected in children [23]. The disease is characterized by periods of unpredictable flares and remissions. IBD afflicts adults and children but the incidence of IBD is increasing in children [24]. Sex differences occur in IBD in terms of, e.g., epidemiological incidence and prevalence [24,25]. Several HRQOL questionnaires have been used in the pediatric IBD population (reviewed in Chen et al., 2017) [26] such as the quality-of-life index for pediatric inflammatory bowel disease (IMPACTIII) which was designed for use in pediatrics, the Transition Readiness Assessment Questionnaire (TRAQ) which assesses health and health care self-management skills that can be used in preparation for transition to adult care [27], the Inflammatory Bowel Disease Questionnaire (IBDQ) and its shorter form, the short inflammatory bowel disease questionnaire (SIBDQ), and the inflammatory bowel disease questionnaire with nine dimensions (IBDQ-9). Most of these questionnaires have multiple domains assessing physical, emotional and social functioning [26]. However, while these are disease-specific questionnaires assessing quality of life in IBD, they are not generic preference-based measures from which health utilities can be derived. With the growth in treatments available for pediatric IBD, cost–utility analyses, the recommended form of economic evaluation, would be aided by the availability of validated preference-based health utility measures for the pediatric population to generate appropriate utilities for IBD health states. This would allow the determination of the universal outcome measure of QALYs to enable comparison of IBD treatments to other disease interventions and other patient groups by decision-makers. Health utility weights for IBD have been examined in very few studies [28,29,30,31] and have not been studied in pediatric IBD populations. Most cost–utility analyses used utilities elicited in a study by Gregor et al. (1997) which evaluated the Standard Gamble (SG), Time Trade Off (TTO), and Visual Analog Scale (VAS) methods to estimate utility scores in Canadian adults with CD [29,31]. Children experience IBD differently than adults, particularly since the disease can affect growth and development [32,33]. Hence, it is likely that children may assign different utility weights to IBD disease states than adults.

The purpose of this study was to further understand the health-related quality of life (HRQOL) in children recently diagnosed with IBD (diagnosed upon study enrolment) and to assess the validity, agreement, and reliability of the CHU9D in pediatric IBD using adult and adolescent tariff sets. This study represents the first to perform a head-to-head comparison of the CHU9D with both adult and youth tariffs, the HUI2 and the HUI3 in a single pediatric cohort with a chronic disease. This study is also the first to examine health utilities in children with inflammatory bowel disease. This research adds to the evidence supporting the use of generic health utility instruments designed for pediatric populations.

## 2. Materials and Methods

### 2.1. Ethics

The study was approved by the Research Ethics Boards of the Hospital for Sick Children in Toronto, ON (#1000039604), the IWK Health Centre in Halifax NS (#1015558), and the Janeway Children’s Health and Rehabilitation Centre in St. John’s NL (#13.229).

### 2.2. Study Design

Participants were recruited from a large, observational cohort of children with newly diagnosed CD and UC from the Canadian Children Inflammatory Bowel Disease Network (CIDsCaNN) study sites (https://cidscann.ca, accessed on 1 February 2021). The CIDsCaNN network includes a patient registry from major IBD centres across Canada that have been studying the pathogenesis and treatment of IBD since 2014. Children were treated at the discretion of the attending clinicians. Three centres from the CIDsCaNN network, a Toronto site, a Halifax site, and a St. John’s site, participated in this study.

### 2.3. Data Collection

Pediatric IBD patients with diagnosed Crohn’s disease or ulcerative colitis from whom parental consent was received or who assented to participating were administered the CHU9D and HUI questionnaires in addition to other the CIDsCaNN study assessments completed at several timepoints following recruitment. Questionnaires were not randomized or administered in a set order. They were administered electronically in English via REDCap and were self-completed or interviewer-assisted. In most cases, all study questionnaires were administered and completed on the same day, at the convenience of the participants. For each participant, the first pair of complete date-matched CHU9D and HUI questionnaires administered following disease diagnosis was analyzed. Incomplete questionnaires were excluded since they could not be appropriately scored with missing values.

### 2.4. Participants

Children were enrolled in the CIDsCaNN network between February 2014 and December 2018. The target population was children with Crohn’s disease or ulcerative colitis aged 6.5 years to 18.1 years. Children below the age of 6 years were excluded as the self-assessed questionnaires were not validated for use in younger children. Children diagnosed with undetermined IBD and those with proxy-completed questionnaires were excluded. Included children did not have other co-morbidities reported outside of their IBD and were enrolled prior to turning 18 years of age. The first CHU9D-HUI paired assessment was administered from 0 to 1735 days (58 months) from the date of diagnosis. A total of 285 children were included in the study, with 199 children diagnosed with CD and 86 children diagnosed with UC.

### 2.5. Questionnaires

The self-complete format of the CHU9D questionnaire was included, and CHU9D proxy-completed questionnaires were excluded as the focus of this study was to ascertain self-reported utility weights. Utility weights for the CHU9D were calculated using Australian adult tariffs based on best–worst scaling and Australian adolescent tariffs based on best–worst scaling [10]. Scoring algorithms were provided in STATA by the developers of the CHU9D [10,11,34]. The STATA algorithms equations were adapted to R and questionnaires were scored using R software (v. 4.0.0) [35].

The HUI questionnaire with a one-week recall period was administered in one of two formats, depending on the preference of the subject or caregiver. One format is self-completed and the other is self-assessed but interviewer-administered. Both formats capture identical information. Questionnaires completed by proxy respondents were excluded. Among participants with CD, 187 (94%) self-completed the questionnaires and 12 were completed by interview. Among participants with UC, 82 (95%) self-completed the questionnaires and 4 were completed by interview. All included CHU9D questionnaires were self-completed. Licenses and scoring algorithms for the HUI were obtained from Health Utilities Inc. (http://healthutilities.com/, accessed on 1 February 2021). Since study subjects were children, the “Fertility” attribute of the HUI2, which deals with the ability to conceive, was omitted in the calculation of HUI2 utilities, as it is optional and not part of the standard questionnaire and its omission does not affect utility scoring [22,36]. The paired HUI2 and HUI3 health utilities were compared to the CHU9D health utilities calculated using the Australian adult tariffs and Australian youth tariffs.

### 2.6. Statistical Analysis

All data analysis was conducted using R statistical packages. Complete cases were used in the analysis, which included completed health assessments related to their IBD and completed CHU9D and HUI questionnaires. Five (2%) of respondents failed to complete the CHU9D questionnaires and 26 (9%) failed to properly complete the HUI questionnaire and were excluded from the analysis. CD and UC patient data were analysed separately. The health status of CD patients and UC patients were assessed by study physicians on the same day (in most cases) or within seven days of questionnaire administration using the weighted Pediatric Crohn’s Disease Activity Index (wPCDAI) [37] and the Pediatric Ulcerative Colitis Activity Index (PUCAI) [38], respectively. Both the wPCDAI and PUCAI provide numerical scores for disease severity based on a composite assessment of physical and physiological symptoms. Where wPCDAI or PUCAI scores were not determined, a physician global assessment (PGA) of disease activity reported as quiescent, mild, moderate, or severe based on history and physical examination was used as a clinical assessment of health status. Descriptive statistics were compiled using the Table One package in R [39]. The Chi-square test was used for categorical variables, the *t*-test was used for continuous variables and the Kruskal–Wallis test was used for non-normally distributed continuous variables for comparisons between sexes. Kruskal–Wallis rank-sum tests were used to compare utilities from the different instruments. Construct validity, agreement and test-retest reliability of the CHU9D were assessed as described below.

#### 2.6.1. Validity

As there is currently no gold standard for the measurement and valuation of health-related quality of life in children, the construct validity of the CHU9D was examined by comparing the health utilities derived from the CHU9D to those derived from the existing HUI2 and HUI3, as these have been used extensively in pediatrics. The Shapiro–Wilk normality test was performed to examine the distribution of the CHU9D utilities calculated with adult and youth tariffs and for the HUI2 and HUI3 health utilities. The null hypothesis was rejected, and normality could not be assumed, based on a *p*-value < 0.05. Spearman correlations were therefore determined between the overall CHU9D scores with adult and youth tariffs, and the overall HUI2 and HUI3 scores.

To further assess construct validity, *a priori* hypotheses regarding expected correlations between domains and attributes measuring similar constructs were postulated and assessed. It was hypothesized that correlations > 0.5 would be observed for conceptually similar domains and correlations in the 0.35 to 0.5 range would be observed for overlapping but conceptually distinct domains. It was hypothesized that r (Spearman rho) > 0.5 for the CHU9D Worry and the HUI2 and HUI3 Emotion domains; the CHU9D Sad and HUI2 and HUI3 Emotion domains; the CHU9D Pain and HUI2 Pain domains; the CHU9D Schoolwork and the HUI2 and HUI3 Cognition domains; the CHU9D Daily Routine and HUI2 Self-care and HUI3 Dexterity domains; the CHU9D Activities and the HUI2 Mobility and the HUI3 Ambulation domains. It was hypothesized that correlation coefficients would fall between 0.35 and 0.5 for the CHU9D Annoyed and HUI2 and HUI3 Emotion domains; the CHU9D Daily Routine and HUI2 Mobility and HUI3 Ambulation domains; the CHU9D Activities and HUI2 Self-care and HUI3 Dexterity domains. To examine which domains had the lowest domain scores within each of the CHU9D, HUI2 and HUI3 instruments, domain scores were ranked from lowest to highest score.

#### 2.6.2. Agreement

The agreement and interchangeability between overall CHU9D scores and the HUI scores were assessed using the Bland–Altman limits of agreement approach [40]. The difference between the utilities for the two approaches was plotted against the mean utilities for the two approaches for each patient’s paired assessment. This determined whether the difference between instruments changed systematically as the mean utility increased or decreased. The range represented by the mean difference ± 1.96 standard deviations (the “limits of agreement”) was plotted. A range within these limits suggest the measures are interchangeable. The Bland–Altman limits of agreement were calculated and plotted using the ‘blandr’ v.0.5.1 R package [41].

#### 2.6.3. Test-Retest Reliability

As CIDsCaNN sites provide ongoing treatment and follow-up, patients had repeat visits approximately every 6 months. A small number of CD and UC patients completed a second set of paired CHU9D and HUI questionnaires and had a stable clinical health status within 6 months of their previous clinic visit. A stable health status was defined as two consecutive assessments of remission, mild, moderate or severe according the wPCDAI score for CD patients and the PUCAI for UC patients. To assess test–re-test reliability, which refers to the inter-rater reliability of utility scores calculated from the same individuals in the same health state at different timepoints, an intraclass correlation coefficient (ICC) was determined from data from questionnaires administered within six months in patients with a stable health status. The coefficient was determined using the R package ‘irr’ v. 0.84.1 using a two-way random effects model with absolute agreement with a 95% confidence interval [42,43].

## 3. Results

### 3.1. Sample Characteristics

A total of 285 children with IBD participated, including 199 children diagnosed with CD and 86 diagnosed with UC. Participant characteristics are listed in Table 1. In the CD group, 36.2% of participants were female and in the UC group, 59.3% of participants were female. In both groups, the mean age of participants was 14 years. In the CD group, 59.8% of participants were of Caucasian origin and in the UC group, 65.1% of participants were of Caucasian origin, with the rest from diverse backgrounds. Participants were recruited predominantly from the Toronto clinical site which started enrolling patients and administering health-related quality-of-life questionnaires earlier than other sites. There were no statistically significant differences in demographic characteristics between males and females for either disease group (Table 1).

The participants’ IBD severity at the time of HRQOL assessment was reported based on the wPCDAI scale for CD patients and the PUCAI scale for UC patients (Table 2). In males with CD, 63.8% were in remission or had mild disease, compared to 63.9% in females with CD. In males with UC, 77.1% were in remission or had mild disease, compared to 72.5% in females with UC. Where wPCDAI scores and PUCAI scores were not available, the PGA was used as a measure of disease status.

A summary of CHU9D health utilities scores calculated using adult and youth tariffs, and HUI2 and HUI3 health utilities for the CD and UC groups are presented in Table 3 and utility ranges are shown in box plots in Supplementary Figures S1 and S2. CHU9D utility scores calculated with youth tariffs were consistently lower than those calculated with adult tariffs in males and females with CD and UC (Table 3). There was a statistically significant difference between males and females with UC in CHU9D utilities with adult and youth tariffs (Kruskall–Wallis tests, *p* = 0.02 for both) but no difference between the sexes in HUI2 and HUI3 utilities (Kruskall–Wallis tests, *p* > 0.05 for both). Box plots comparing overall utilities between the different health utility instruments for CD and UC are shown in Supplemental Figures S1 and S2 respectively. Overall, CHU9D utilities with youth tariffs were lower compared to the CHU9D utilities with adult tariffs, the HUI2 utilities and the HUI3 utilities in CD and UC. In CD, there was a significant difference between the overall CHU9D (with both tariff sets) and HUI2 utilities and between the CHU9D (with both tariff sets) and HUI3 utilities (Kruskall–Wallis test *p* < 0.0001). In UC, there was a significant difference between overall CHU9D (with adult tariffs and youth tariffs) and HUI2 utilities (*p* = 0.004), and between overall CHU9D (with adult and youth tariffs) and HUI3 utilities (*p* = 0.03). There was also a significant difference between the HUI2 and HUI3 utilities in CD and UC (*p* < 0.00001 and *p* = 0.004, respectively). There was a significant difference between the CHU9D adult tariff utilities and youth tariff utilities in CD (*p* = 0.002), but not between tariff sets in UC at the 95% confidence level.

### 3.2. Validity

The correlations of CHU9D utilities with HUI2 and HUI3 utilities for CD and UC groups are displayed in Table 4. The CHU9D showed a moderate to strong significant correlation (at *p* < 0.05) with the HUI2 and HUI3 scores. In CD, the correlation between the CHU9D with adult tariffs and the HUI2 and the HUI3 was 0.65 and 0.62, respectively (Table 4). In CD, the correlation between the CHU9D with youth tariffs and the HUI2 and the HUI3 was 0.67 and 0.65, respectively (Table 4). In UC, the correlation of the CHU9D with HUI2 and HUI3 scores was 0.67 with adult tariffs, and was 0.69 with youth tariffs. The HUI2 and HUI3 scores were, as expected, strongly correlated with r = 0.85 in CD and 0.87 in UC (*p* < 0.05).

Spearman correlations between conceptually similar domains are presented in Table 5. The Pain domain was the only domain that was strongly correlated between the CHU9D and HUI2 and HUI3 assessments. In CD, the correlation between the CHU9D and HUI2 Pain domains was 0.65 with adult tariffs and 0.66 with youth tariffs (*p* < 0.05), and was 0.66 and 0.70 between the HUI3 and CHU9D adult and youth tariffs, respectively (*p* < 0.05) (Table 5). In UC, the correlation between the CHU9D and HUI2 Pain domains was 0.53 with adult tariffs and 0.61 with youth tariffs (*p* < 0.05), and was 0.61 and 0.67 between the HUI3 and CHU9D adult and youth tariffs, respectively (*p* < 0.05) (Table 5). Other conceptually overlapping domain pairs that met *a priori* hypotheses (r = 0.35 to 0.5) regarding strength of correlation were the CHU9D Annoyed domain and the HUI Emotion domain with r = 0.44 and r = 0.40 between CHU9D and HUI2 domain utilities in CD and UC, respectively, and with r = 0.40 and r = 0.37 between CHU9D and HUI3 domain utilities in CD and UC, respectively (*p* < 0.05) (Table 5). Contrary to the expected strong correlation between the pairs, the CHU9D Sad domain and HUI Emotion domains were not strongly correlated.

To gain insight into whether there were differences in which domains were scored the lowest between the different health utility instruments, each domain score was ranked from lowest (worst) to highest (best) for each instrument (Table 6). The mean multi-attribute (rather than single) utility scores were calculated for each domain in the HUI2 and HUI3 for all participants with CD and UC, respectively, and the mean scores for each CHU9D domain were calculated. The domain means were then ranked from lowest to highest. For the CHU9D with adult tariffs, HUI2 and HUI3 instruments, the Pain domain was ranked the lowest. This was expected, since IBD patients can experience significant pain during the course of their disease. Interestingly, for the CHU9D with youth tariffs, Sleep was ranked the lowest. This is not unexpected, since IBD can affect sleep greatly due to multiple bathroom trips during the night, pain, or anxiety over the disease.

### 3.3. Agreement

**Figure 1:**
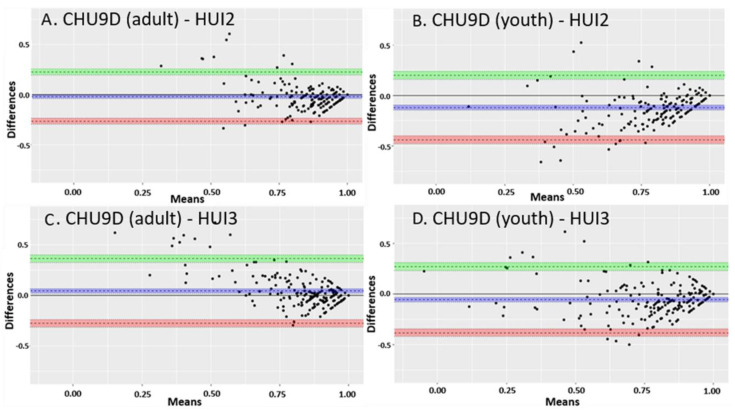
Bland–Altman limits of agreement plots between CHU9D (adults and youth tariffs) and HUI2 and HUI3 utility scores in children with Crohn’s disease. (**A**) The mean difference between the CHU9D scores with adult tariffs and HUI2 scores. (**B**) The mean difference between the CHU9D scores with youth tariffs and HUI2 scores. (**C**) The mean difference between the CHU9D scores with adult tariffs and HUI3 scores. (**D**) The mean difference between the CHU9D scores with youth tariffs and HUI3 scores. The dotted line close to zero and the purple shading represent the mean difference between the CHU9D and HUI scores. The upper limit of the 95% confidence interval is represented by the dotted line above zero and the green shading. The lower limit of the 95% confidence interval is represented by the dotted line below zero and the orange shading.

**Figure 2:**
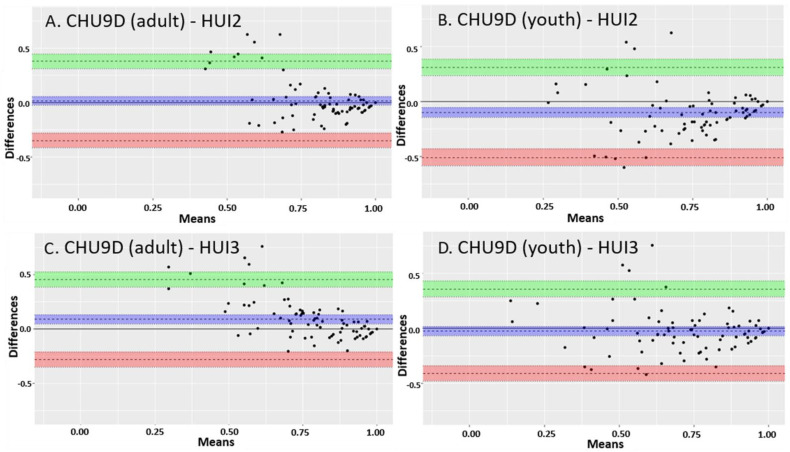
Bland–Altman limits of agreement plots between CHU9D (adults and youth tariffs) and HUI2 and HUI3 utility scores in children with ulcerative colitis. (**A**) The mean difference between the CHU9D scores with adult tariffs and HUI2 scores. (**B**) The mean difference between the CHU9D scores with youth tariffs and HUI2 scores. (**C**) The mean difference between the CHU9D scores with adult tariffs and HUI3 scores. (**D**) The mean difference between the CHU9D scores with youth tariffs and HUI3 scores. The dotted line close to zero and the purple shading represents the mean difference between the CHU9D and HUI scores. The upper limit of the 95% confidence interval is represented by the dotted line above zero and the green shading. The lower limit of the 95% confidence interval is represented by the dotted line below zero and the orange shading.

There was moderate agreement between the CHU9D scores and the HUI2 and the HUI3 scores (Figure 1 and Figure 2). There was greater agreement for higher utilities scores (greater than 0.75) than lower ones. In children with CD, the mean difference between the CHU9D overall scores with adult tariffs and HUI2 scores was −0.021, 95% CI (−0.038, −0.003). The mean difference between the CHU9D scores with youth tariffs and HUI2 scores was −0.120, 95% CI (−0.143, −0.097). The mean difference between the CHU9D scores with adult tariffs and HUI3 scores was 0.043, 95% CI (0.020, 0.066). The mean difference between the CHU9D scores with youth tariffs and HUI3 scores was −0.056, 95% CI (−0.079, −0.032). In children with UC, the mean difference between the CHU9D scores with adult tariffs and HUI2 scores was 0.015, 95% CI (−0.025, 0.055). The mean difference between the CHU9D scores with youth tariffs and HUI2 scores was −0.100, 95% CI (−0.144, −0.055). The mean difference between the CHU9D scores with adult tariffs and HUI3 scores 0.088, 95% CI (0.048, 0.128). The mean difference between the CHU9D scores with youth tariffs and HUI3 scores −0.027, 95% CI (−0.069, 0.015). The mean difference between CHU9D scores with adult tariffs and youth tariffs was 0.115, 95% CI (0.094, 0.135) and 0.099, 95% CI (0.087, 0.111) in CD and UC, respectively (plot not shown). Interestingly, the mean difference between the CHU9D scores with adult and youth tariffs was greater than the mean difference between the CHU9D utilities with adult tariffs and HUI2 in CD and UC. In both CD and UC, the absolute value of the difference between the CHU9D with adult tariffs and the HUI2 scores were the lowest, indicating the greatest agreement between these two instruments. In both populations, the majority of assessments were in patients in remission, which could partially explain why there was greater agreement for higher utility scores.

### 3.4. Test–Retest Reliability

Eleven CD participants and 9 UC participants completed a second set of paired CHU9D and HUI questionnaires and had a stable health state within 6 months of their previous clinical visit. Of the 11 CD participants, 8 were in remission and 3 were categorized as having mild disease at the time of each assessment. Of the 9 CD participants, 8 were in remission and 1 was categorized as having moderate disease at the time of each assessment. The mean assessment intervals were 129 days (s.d. 42.5) and 155 days (s.d. 22.4) for CD and UC participants, respectively. In the CD participants, all health utility instruments showed a good test–retest, reliability with ICC coefficients ranging from 0.71 to 0.89 with a *p*-value < 0.05 (Table 7). However, in UC participants, all instruments showed poor test–retest reliability, with ICCs ranging from −0.08 to 0.48, and none were significant. Negative ICC values may be a result of a high mean square error and suggest poor agreement [44]. Given that so few stable participants completed a second set of questionnaires and the long time-interval between questionnaires, these results should be considered exploratory. As this was an observational study and not a strict protocol, the number of participants coming in for clinic visits with a short time interval between visits could not be anticipated.

## 4. Discussion

### 4.1. Health-Related Quality of Life in Pediatric IBD

Health utilities were generated in children with CD and UC using the CHU9D and HUI generic preference-based measures. The sample population included children experiencing inactive (in remission) to strongly active disease with a greater proportion of children in remission. Despite a greater proportion of children being in remission, health utilities varied widely regardless of the health utility instrument used and some children had low health utilities even with little or no physical symptoms of disease. In addition, an obvious trend toward a lower utility associated with greater disease severity could not be established. This suggests that each child with IBD can have a very different quality of life regardless of their disease severity at any given time. Considering that most children with IBD are dealing with a chronic, unpredictable affliction with no known cure and a chronic treatment regimen even during remission, heterogeneity in HRQOL scores could be expected. For example, some children may experience more anxiety during periods of remission than others, which could affect their utility scores. To gain a greater understanding of the value of generic preference-based HRQOL measures in pediatric IBD, comparisons between generic measures and disease-specific health scores should be undertaken in future studies. Largely due to nutritional deficiencies, IBD can affect growth [32,33] and may affect children of different ages and different stages of growth differently, such as those before and after puberty. Health utility instruments developed for children should be able to capture these differences.

The study results show similar health utility scores between health utility instruments used. For example, in CD males, the mean utility scores were 0.8846, 0.7730, 0.8846, 0.8206 for CHU9D with adult tariffs, CHU9D with youth tariffs, HUI2 and HUI3, respectively. However, there was a statistically significant difference between utilities from the different tariffs and health utility instruments, suggesting the instruments are not interchangeable, and the choice of instrument could impact economic evaluation results. The differences between health utilities elicited with different instruments in the same population was also observed by Gregor et al. (1997) in CD adults [29]. Gregor et al. (1997) observed mean utilities of 0.92 with the TTO method, 0.81 with the SG method and 0.71 with the VAS method in adults with active and inactive CD [29]. Based on a meta-analysis, Malinowski and Kawalec (2016) estimated the mean health utility of UC patients to be 0.8726, 95% CI (0.8457, 0.8995) in remission and 0.6992, 95% CI (0.5847, 0.8136) in active disease, and estimated the mean health utility of CD patients as 0.8403, 95% CI (0.8012, 0.8794) in remission and 0.7533, 95% CI (0.6887, 0.8178) in active disease [31]. The results in the present study are similar to results from adult studies. Based on these values, a range of health utilities to describe the same disease can be expected and may depend on the method of utility elicitation. A variation due to elicitation methods was also demonstrated by Whitehurst et al., 2014 in a study examining contemporaneous EQ-5D and SF-36 responses [45]. This variation in health utilities should be taken into account by those conducting cost–utility analyses in IBD. It should also be noted that, due to the variation in utilities in IBD, a difference as small as 0.03 or more may not be clinically meaningful, as has been suggested for the HUI [46].

The results of this study showed that overall, health utility scores were similar between children with CD and UC with the average of the mean utilities being 0.8; however, in UC, mean utilities were lower, ranging from 0.717 to 0.832, and in CD they ranged from 0.754 to 0.874 with the different health utility instruments (Supplementary Figures S1 and S2). There was a statistically significant difference in CHU9D scores between males and females with UC, but not in CD or in HUI2 and HUI3 scores. Epidemiological and clinical differences between the sexes have been reported in IBD [24,25,47]. However, this may also be a result of the domains described by the CHU9D compared to the HUI2 and HUI3. This paper is the initial report of research examining the use of the CHU9D in pediatric IBD. Additional research to further examine utilities by health states categorized with clinical assessment measures in CD and UC is warranted.

### 4.2. Validity

With no gold standard for health-related quality of life available, construct validity was estimated by comparison with the longstanding HUI. There was a moderately strong correlation between the CHU9D, HUI2 and HUI3 in the pediatric IBD population indicating good construct validity of the CHU9D scored with either tariff set. Paired comparisons of the CHU9D scores with adult and youth tariffs showed that the mean and median of youth tariffs were lower than adult tariffs, suggesting that adolescents weight health states differently than adults. This phenomenon has been previously reported by Ratcliffe et al. (2012, 2016), and has been attributed to a stronger weighting of mental health impairments, reflected in the worried, sad and annoyed domains, by adolescents compared to a stronger weighting of physical impairment (pain) by adults [10,48]. Interestingly, in the current study, when each domain score was ranked from lowest to highest, there was a difference in which domains were ranked the lowest between the CHU9D adult and youth tariffs. Using the CHU9D youth tariffs, the Sleep domain was given the lowest score, followed by the Annoyed and Daily Routine domains in CD and the Daily Routine and Annoyed domains in UC. Using CHU9D adult tariffs, the Pain domain was ranked as the lowest scoring domain, which was also the lowest scoring domain in the HUI2 and HUI3 assessments. Following the Pain domain, were the Tired and Sleep domains, with CHU9D using adult tariffs, and the Emotion domains with the HUI2 and HUI3. This may also explain why the CHU9D adult tariffs utility scores were more in agreement with the HUI2. The example of different utility results from the different tariff sets in pediatric IBD supports the use of adolescent or pediatric tariffs to assess a HRQOL reflective of the pediatric experience.

With regard to agreement, there was greater agreement between paired CHU9D and HUI scores with higher utilities than lower utilities based on the Bland–Altman limits of agreement results. This pattern was also seen in Ratcliffe et al., 2012, in a comparison between the CHU9D with adolescent tariffs and the HUI2 which also claimed a moderate agreement between the CHU9D and HUI2 utilities [49]. This may be due to the fact that there were more participants with milder disease reflecting higher utilities. This may also be due to lower discriminant validity and a larger variation in utilities in more severely affected participants. While the lack of agreement at lower utilities may lead one to discount the validity of the CHU9D, it is important to consider the prevalence of lower utilities. To interpret the disagreement at lower utilities in the Bland–Altman Agreement plots, it is important to note that there were too few participants with low utility scores to conclusively determine whether there was disagreement at lower utilities. It is also noteworthy that the same pattern of agreement was seen in other studies [49]. It is also important to note that low utility scores, particularly those below 0.5, are considered very low and have rarely been reported for any pediatric disease [50]. Hence, it may not be possible to achieve low utilities in a large enough subset of pediatric IBD patients to thoroughly examine agreement or disagreement at low utility levels. Therefore, while there was disagreement between the instruments at low utility scores, the high level of agreement at higher utility scores should be considered supportive of the validity of the CHU9D. A more thorough examination of health utilities and health states in pediatric IBD requires further research.

There was good test–retest reliability in CD participants but poor test–retest reliability in UC participants. The number of stable patients was too few to provide a robust assessment of test–retest reliability, and, as such, should be considered exploratory. Additional studies of test–retest reliability should be conducted with a greater number of participants in a stable health state and with a shorter interval of time between questionnaire administrations. However, another explanation may be that UC participants experience more fluctuation in quality of life even when their symptoms are relatively constant.

### 4.3. Strengths and Limitations

A strength of this study was the head-to-head comparison between the CHU9D and the HUI in IBD. This allowed the direct comparison of the CHU9D and HUI in the same population and an assessment of the performance of the CHU9D.

Another strength was the direct comparison of the CHU9D utilities from adult and youth tariff sets, where both tariff sets were derived using the same method (best–worst scaling) and in the same nation (Australia) [10]. Comparing utilities from tariffs elicited from the same method reduces potential confounding of results due to different methods of elicitation, such as in the case when adult UK tariffs elicited using the SG method were compared to the CHU9D Australian adolescent tariffs elicited using best–worst scaling [10].

This study also offered an opportunity to compare results between CD and UC pediatric populations recruited from the same clinical sites. The study identified differences in total health utilities and in individual domain scores between pediatric CD and UC. It also identified a potentially meaningful difference between CHU9D utilities between males and females with UC. This stresses the importance of further study of sex and gender differences within health utility research and that utilities assigned to a health state may not be sex- or gender-neutral.

A major limitation to the assessment of test–retest reliability and responsiveness of the CHU9D was the long intervals between repeat administrations of HRQOL questionnaires, often exceeding 6 months. While there were 199 participants with CD and 86 with UC, there were too few participants with documented stable and unstable health states between patients to enable an assessment of responsiveness of the CHU9D. As CIDsCaNN was an observational clinical study, patients were treated at the discretion of the physician and, if they did not need in-person physician follow-up within short periods of time because they were in remission or recovering as expected on treatment, an in-person clinic site visit and administration of a HRQOL questionnaire was not conducted. It was assumed that all participants were receiving treatment according to standard clinical practice guidelines. As this was an observational study and not a strict protocol, the number of participants coming in for clinic visits with a short time interval between visits could not be anticipated.

Another limitation to assessing CHU9D performance characteristics was that the health of the participants was skewed towards the majority who were in remission. Therefore, a more balanced HRQOL assessment of participants in remission, mild, moderate or severe disease could not be assessed. Nevertheless, the fact that some participants in remission exhibited low health utility suggests that IBD can result in a lower HRQOL even in the absence of physical symptoms. Low utility may have been exhibited in children in remission because of the fatigue and emotional factors and anxiety caused by IBD which may not be captured in the clinical assessment. Future studies should include a more balanced population of patients in active and inactive disease.

### 4.4. Challenges in Assessing Health Utilities in Children

The lower utilities with the youth tariffs of the CHU9D suggest that children give different weights to certain states within domains than adults, which can result in lower domain scores with the potential to affect the results of a cost–utility analysis. Hence, assessing health utilities in children using tariffs from children is preferred. However, this study also illustrated several challenges in assessing health utilities in children. Due to a paucity of prospective randomized controlled clinical trials with children that include economic evaluations, pediatric research related to economic evaluation often relies on retrospective analysis of observational studies. Treatment and patient visits are often scheduled as-needed or at the discretion of the clinician. As a result, the timely administration of quality-of-life questionnaires that correspond to particular health states may not occur.

Another challenge is deciding on the elicitation method most feasible for children. This study and others have shown that variation in tariffs and elicitation methods results in different health utilities which can affect the results of cost–utility analyses and, ultimately, could affect health funding decisions [50,51]. The lack of health utility instruments validated for use in children younger than seven poses challenges in collecting quality-of-life information in children afflicted by early onset of disease. Parent proxy questionnaires may not be a good representation of the pediatric experience, particularly for very young children [52].

As this study was the first to examine health utilities in children with IBD, results were aggregated for all age groups. Future work could include a sub-analysis between adolescents and younger children with IBD to examine whether stages of growth exhibit different HRQOL scores. In addition, an examination of known-groups validity using established parameters of disease severity and corresponding utilities can be examined to determine health utilities describing health states in pediatric IBD.

## 5. Conclusions

Considering the overall correlations, and agreement at high utility scores most often observed in IBD, the CHU9D showed good construct validity with the HIU2 and HUI3 in a CD and UC population of children aged 7 to 18 years. The CHU9D, HUI2 and HUI3 utilities in pediatric IBD were similar to utilities derived in adults. There was a large range of health utilities for each health utility instrument in CD and UC indicating a heterogeneous HRQOL experience in children with IBD. CHU9D health utilities calculated with youth tariffs were lower than those calculated using adult tariffs, supporting the need for additional health utility and quality-of-life research in childhood disease. Additional studies in other pediatric illnesses with head-to-head comparisons of utilities derived with youth tariffs and adult tariffs may serve to illustrate differences between the pediatric and adult experience. Quality-of-life studies in children of different age groups can shed light on differences between very young children and those transitioning to adulthood.

## Figures and Tables

**Table 1:** Patient characteristics stratified by sex.

Characteristic	CD		UC	
	Males	Females	Males	Females
***n***	127	72	35	51
**Mean age, years (SD)**	13.8 (2.3)	14.2 (2.5)	14.2 (2.4)	13.9 (3.1)
**Age range (minimum to maximum, years)**	7.3–17.8	7.6–18.1	9.4–18.0	6.5–18.1
**Ethnicity (%)**				
Caucasian	73 (57.5)	46 (63.9)	25 (62.9)	34 (66.7)
Mixed	13 (10.2)	7 (9.7)	2 (5.7)	4 (7.8)
South Asian	14 (11.0)	2 (2.8)	7 (20.0)	7 (13.7)
East and Southeast Asian	5 (3.9)	1 (1.4)	0 (0.0)	1 (2.0)
Caribbean, Latin, Central or South American	3 (2.4)	5 (6.9)	0 (0.0)	1 (2.0)
West Central Asian and Middle Eastern	2 (1.6)	2 (2.8)	1 (2.9)	3 (5.9)
North, Southern, East, Central or West African	5 (3.9)	2 (2.8)	2 (5.7)	1 (2.0)
Other or Unknown	12 (9.4)	7 (9.7)	1 (2.6)	0 (0.0)
**Clinical Site**				
Toronto	98 (77.2)	54 (75.0)	30 (85.7)	44 (86.3)
Halifax	29 (22.8)	15 (20.8)	5 (14.3)	6 (11.8)
St. John’s	0 (0.0)	3 (4.2)	0 (0.0)	1 (2.0)

Demographics of patients that completed at least one date-matched CHU9D and HUI paired assessment stratified by sex. There were no significant differences between the sexes (*p* < 0.05). Age at CHU9D and HUI completion is reported. CD = Crohn’s disease, UC = ulcerative colitis, SD = standard deviation.

**Table 2:** The inflammatory bowel disease health status of participants at the time of health-related quality of life assessment.

Characteristic	CD		UC	
	Males	Females	Males	Females
***n***	127	72	35	51
**wPCDAI or PUCAI Score mean (SD)**	22.6 (24.0) Mild	30.4 (29.4) Mild	17.2 (24.2) Mild	19.2 (26.4) Mild
**wPCDAI or PUCAI Score median (IQR)**	15.0 (37.5) Mild	25.0 (40.0) Mild	5.0 (23.8) Remission	5.0 (30.0) Remission
**wPCDAI or PUCAI** **Range (minimum to maximum)**	0.0–105.0 (Remission to Severe)	0.0–110.0 (Remission to Severe)	0–80.0 (Remission to Severe)	0–80.0 (Remission to Severe)
**wPCDAI Category or PUCAI Category** ***n* (%)**				
Remission	51 (40.2)	24 (33.3)	18 (51.4)	28 (54.9)
Mild	30 (23.6)	22 (30.6)	9 (25.7)	9 (17.6)
Moderate	13 (10.2)	4 (5.6)	3 (8.6)	7 (13.7)
Severe	11 (8.7)	13 (18.1)	4 (11.4)	5 (9.8)
Missing (Unknown)	22 (17.3)	9 (12.5)	1 (2.9)	2 (3.9)

For Crohn’s disease (CD), the weighted Pediatric Crohn’s disease Activity Index (wPCDAI) was used to assess severity and for ulcerative colitis (UC), the Pediatric Ulcerative Colitis Activity Index (PUCAI) was used. wPCDAI scores less than 12.5 indicate remission, between 12.5 to 40 indicate mild disease, between 40 to 57.5 indicate moderate disease and scores greater than 57.5 indicate severe disease PUCAI scores less than 10 indicate remission, between 10 to 34 indicate mild disease, between 35 to 64 indicate moderate disease and scores between 65 to 85 indicate severe disease. There were no statistically significant differences in scores between the sexes, at *p* < 0.05. SD = standard deviation, IQR = interquartile range.

**Table 3:** CHU9D and HUI health utilities at first paired assessment for CIDsCaNN Crohn’s disease (CD), and ulcerative colitis (UC) participants stratified by sex.

			CHU9D Utility (Adult Tariffs)	CHU9D Utility (Youth Tariffs)	HUI2 Utility	HUI3 Utility
CD	Males (*n* = 127)	Mean (SD)	0.862 (0.122)	0.773 (0.203)	0.885 (0.152)	0.821 (0.220)
		Median (IQR)	0.880 (0.148)	0.810 (0.232)	0.926 (0.136)	0.879 (0.230)
		Range (minimum to maximum)	0.380–1.000	0.052–1.000	0.174–1.000	−0.160–1.000
	Females (*n* = 72)	Mean (SD)	0.838 (0.112)	0.721 (0.200)	0.855 (0.155)	0.791 (0.216)
		Median (IQR)	0.866 (0.163)	0.757 (0.274)	0.907 (0.177)	0.846 (0.224)
		Range (minimum to maximum)	0.558–1.000	0.167–1.000	0.266–1.000	0.081–1.000
UC	Males (*n* = 35)	Mean (SD)	0.869 * (0.115)	0.778 * (0.210)	0.854 (0.200)	0.799 (0.212)
		Median (IQR)	0.885 (0.154)	0.810 (0.311)	0.937 (0.189)	0.879 (0.264)
		Range (minimum to maximum)	0.567–1.000	0.230–1.000	0.212–1.000	0.232–1.000
	Females (*n* = 51)	Mean (SD)	0.806 * (0.129)	0.675 * (0.216)	0.791 (0.214)	0.706 (0.251)
		Median (IQR)	0.826 (0.174)	0.686 (0.296)	0.868 (0.222)	0.748 (0.307)
		Range (minimum to maximum)	0.480–1.000	0.174–1.000	0.257–1.000	0.011–1.000

* *p* = 0.02 (statistically significant difference between sexes), SD = standard deviation, IQR = interquartile range.

**Table 4:** Spearman correlation of CHU-9D with HUI overall utilities.

	CD		UC	
	*N* = 199		*N* = 86	
	CHU-9D with Adult Tariffs	CHU-9D with Youth Tariffs	CHU-9D with Adult Tariffs	CHU-9D with Youth Tariffs
HUI2	0.65 *	0.67 *	0.67 *	0.69 *
HUI3	0.62 *	0.65 *	0.67 *	0.69 *

N refers to the number of paired assessments where there was one pair per participant. * *p* < 0.05 for all correlations indicating significant correlation between the assessments. CD = Crohn’s disease, UC = ulcerative colitis.

**Table 5:** CHU9D, HUI2 and HUI3 domain correlations for anticipated similar domains.

CHU9D Domains	HUI2 Domains					HUI3 Domains				
	Emotion	Cognition	Mobility	Self-Care	Pain	Emotion	Cognition	Ambulation	Dexterity	Pain
**Worry**	0.43					0.30				
	0.37					0.21				
**Sad**	0.38					0.42				
	0.47					0.33				
**Pain (adult tariff/youth tariff)**					**0.65/0.66**					**0.66/0.70**
					**0.53/0.61**					**0.61/0.67**
**Annoyed**	**0.44**					**0.40**				
	**0.40**					**0.37**				
**Schoolwork (adult tariff/youth tariff)**		0.22/0.23					0.24			
		0.32					0.3			
**Daily Routine**			0.31	0.26				0.33	−0.04 *	
			0.12	0.41				0.12 *	−0.01 *	
**Activities**			0.30	0.29				0.33	0.10 *	
			0.36	**0.4**				0.36	0.15 *	

* indicates that results were not statistically significant, *p* > 0.05. Where youth tariff correlations were different than adult tariff values, the Spearman rho (r) for youth tariffs is indicated in parentheses. Domains not shown for the HUI2 and HUI3 were not tested. Bold indicates correlation coefficients that agreed with the *a priori* hypothesis. Lighter shades (upper values in a cell) refer to Crohn’s disease; darker shades or lower values in a cell refer to ulcerative colitis. Similar-coloured domains indicate overlapping domains.

**Table 6:** The domain rankings with each health utility instrument.

	CHU9D with Adult Tariffs		CHU9D with Youth Tariffs		HUI2		HUI3	
	CD	UC	CD	UC	CD	UC	CD	UC
Lowest scoring domain	Pain	Pain	Sleep	Sleep	Pain	Pain	Pain	Pain
	Tired	Tired	Annoyed	Daily Routine	Emotion	Emotion	Emotion	Emotion
	Sleep	Sleep	Daily Routine	Annoyed	Sensation	Sensation	Cognition	Cognition
	Annoyed	Annoyed	School	Pain	Cognition	Cognition	Vision	Ambulation
	Sad	Sad	Pain	School	Self-care	Mobility	Ambulation	Vision
	Worry	Worry	Tired	Tired	Mobility	Self-care	Speech	Dexterity
	School	Daily Routine	Activities	Activities			Hearing	Speech
	Daily Routine	School	Sad	Sad			Dexterity	Hearing
Highest scoring domain	Activities	Activities	Worry	Worry				

The mean score was determined for each domain and ranked from lowest (worst) to highest (best). CD = Crohn’s disease, UC = ulcerative colitis. Note the different ranks between the CHU9D adult and youth tariffs.

**Table 7:** Test–retest reliability based on intra-class correlation in 11 stable Crohn’s disease (CD) and 9 ulcerative colitis (UC) participants.

Instrument	Intraclass Correlation (95% CI) in CD Based on wPCDAI Health Status (*n* = 11)	Intraclass Correlation (95% CI) in UC Based on PUCAI Health Status (*n* = 9)
CHU9D (adult tariffs)	0.844 (0.54, 0.955) *	0.455 (−0.174, 0.839)
CHU9D (youth tariffs)	0.889 (0.613, 0.97) *	0.476 (−0.154, 0.847)
HUI2	0.706 (0.223, 0.911) *	−0.0783 (−0.643, 0.573)
HUI3	0.886 (0.587, 0.969) *	−0.103 (−0.692, 0.567)

Values less than 0.5 are indicative of poor reliability, values between 0.5 and 0.75 indicate moderate reliability, values between 0.75 and 0.9 indicate good reliability, and values greater than 0.90 indicate excellent reliability. For the correlations, * indicates *p*-value < 0.05. SD = standard deviation, wPCDAI = weighted Pediatric Crohn’s Disease Activity Index, PUCAI = Pediatric Ulcerative Colitis Activity Index.

## Data Availability

Informed consent was obtained from all subjects and/or parents of subjects involved in the study.

## Supplementary Materials

The following are available online at https://www.mdpi.com/article/10.3390/children8050343/s1, Figure S1: Box plot of utilities from the CHU9D, HUI2 and HUI3 health utility instruments in children with CD; Figure S2. Box plot of utilities from the CHU9D, HUI2 and HUI3 health utility instruments in children with UC.
