# Supplementary material for: An Assessment of the Validity and Reliability of the Pediatric Child Health Utility 9D in Children with Inflammatory Bowel Disease
Source: Children (Basel). 2021 Apr 27;8(5):343. doi: 10.3390/children8050343 (PMC8146594; doi:10.3390/children8050343)
Supplement: Supplementary file 1 [file children-08-00343-s001.zip › children-1142892-supplementary.pdf]

Supplementary Materials:

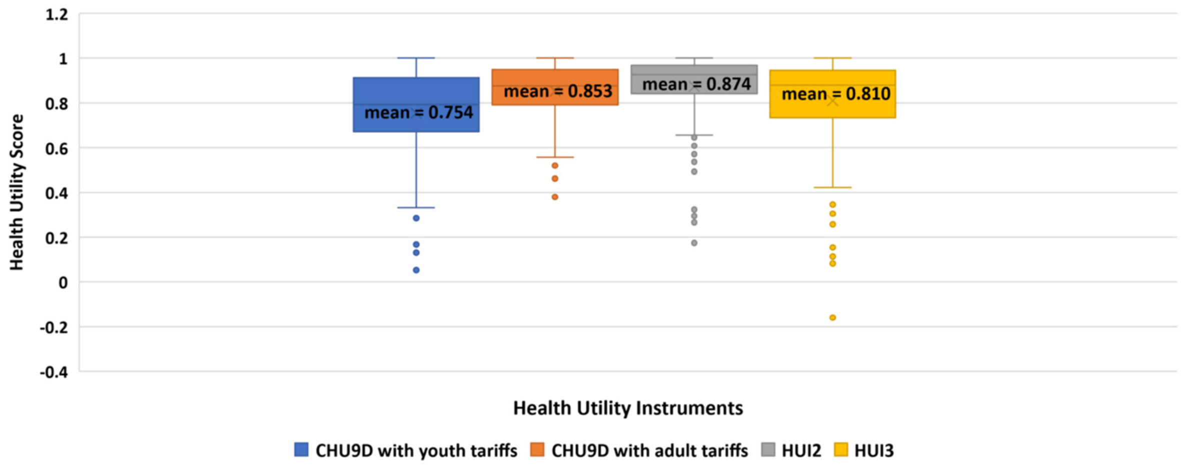

Figure S1. Box plot of utilities from the Child Health Utility 9D (CHU9D), Health Utility Index Mark 2 (HUI2) and Health Utility Index Mark 3 (HUI3) health utility instruments in children with Crohn's disease.

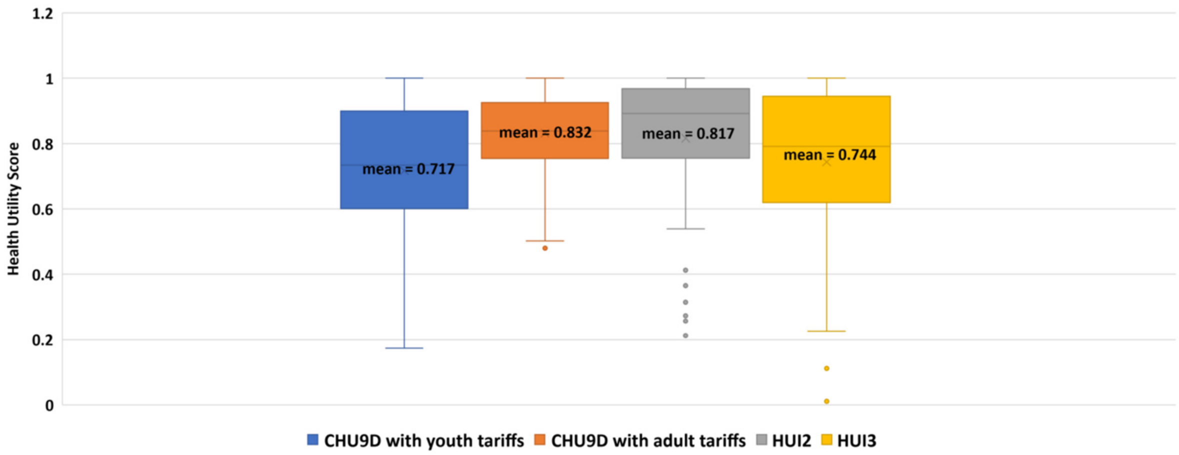

Figure S2. Box plot of utilities from the Child Health Utility 9D (CHU9D), Health Utility Index Mark 2 (HUI2) and Health Utility Index Mark 3 (HUI3) health utility instruments in children with ulcerative colitis.
